# Unlocking the potential of publicly available microarray data using inSilicoDb and inSilicoMerging R/Bioconductor packages

**DOI:** 10.1186/1471-2105-13-335

**Published:** 2012-12-24

**Authors:** Jonatan Taminau, Stijn Meganck, Cosmin Lazar, David Steenhoff, Alain Coletta, Colin Molter, Robin Duque, Virginie de Schaetzen, David Y Weiss Solís, Hugues Bersini, Ann Nowé

**Affiliations:** 1 AI (CoMo), Vrije Universiteit Brussel, 1050 Brussels, Pleinlaan 2, Belgium; 2 IRIDIA, Université Libre de Bruxelles, Avenue F. D. Roosevelt 50, 1050 Brussels, Belgium

**Keywords:** Batch effect removal, Data integration, Gene expression, Microarray repositories, InSilico DB, Reproducibility

## Abstract

**Background:**

With an abundant amount of microarray gene expression data sets available through public repositories, new possibilities lie in combining multiple existing data sets. In this new context, analysis itself is no longer the problem, but retrieving and consistently integrating all this data before delivering it to the wide variety of existing analysis tools becomes the new bottleneck.

**Results:**

We present the newly released inSilicoMerging R/Bioconductor package which, together with the earlier released inSilicoDb R/Bioconductor package, allows consistent retrieval, integration and analysis of publicly available microarray gene expression data sets. Inside the inSilicoMerging package a set of five visual and six quantitative validation measures are available as well.

**Conclusions:**

By providing (i) access to uniformly curated and preprocessed data, (ii) a collection of techniques to remove the batch effects between data sets from different sources, and (iii) several validation tools enabling the inspection of the integration process, these packages enable researchers to fully explore the potential of combining gene expression data for downstream analysis. The power of using both packages is demonstrated by programmatically retrieving and integrating gene expression studies from the InSilico DB repository [https://insilicodb.org/app/].

## Background

An increasing amount of gene expression data sets is becoming available through public repositories (e.g., NCBI GEO [1,2], ArrayExpress [3]). All this new information offers potential clues for treatment of different diseases but one of the current challenges in the field is to unlock the potential of this data. Combining a large number of gene expression data sets originating from different labs could be beneficial for the discovery of new biological insights and could increase the statistical power of gene expression analysis, but then this data should be combined in a consistent manner.

The first hurdle for large-scale gene expression analysis is obtaining access to consistently annotated and preprocessed data [4]. Inside the inSilico Project^1^ the inSilicoDb R/Bioconductor package was released [5], offering programmatical access to all expertly-curated and uniformly preprocessed expression profiles from the InSilico DB repository [6].

However, accessing uniformly represented data is only the first step when combining and integrating gene expression data sets since the use of different experimentation plans, platforms and methodologies by different research groups introduces undesired batch effects in the gene expression measurements [7] thus severely hindering downstream analysis. The problems raised by the batch specific unwanted variation as well as the potential sources leading to batch effects have already been revealed and widely discussed in a number of publications l [8-14]. It is clear that it is necessary to incorporate the adjustment for batch effects as a standard step in the analysis of high-throughput data [15] and several methods to remove this specific type of bias - while preserving the biological variance - have been proposed these last years [16-19]. Unfortunately, there is no single *best* method yet that can be used in all situations.

We present here the inSilicoMerging R/Bioconductor package, which combines several of the most used methods to remove the unwanted batch effects in order to combine, or *merge* different data sets in an intuitive and user-friendly manner.

Evaluating and validating the results of batch effect removal methods is perhaps as important as the batch effect removal process itself. Without good and reliable evaluation tools, these methods could result in an even increased distortion of the data, introducing serious errors in the results of any downstream analysis performed. Therefore, five simple but powerful visual inspection tools and six quantitative measures to evaluate the different batch-effect removal methods, are provided as well.

Although this package can be used as a stand-alone tool, its power lies in combining it with tools like the inSilicoDb package, thereby paving the way towards large-scale meta-analysis of gene expression repositories.

## Implementation

The inSilicoMerging package is part of the open-source Bioconductor [20] project since release 2.10.

### Data formats

A conscious choice has been made to work with the internal data formats of Bioconductor for handling gene expression data objects, namely ExpressionSets. Each function can take as input a list of ExpressionSet objects and the returned value of the different merging methods is a single ExpressionSet, containing the merged data.

### Link with InSilico DB

There is a programmatic link with the InSilico DB genomics data hub [6] through the R/Bioconductor inSilicoDb package [5], providing uniformly curated and preprocessed gene expression data. All genomic data sets are formatted as fully annotated ExpressionSets which can be used immediately within the inSilicoMerging package. Examples in the next section will illustrate this seamless integration.

On the browse page of InSilico DB [https://insilicodb.org/app/browse] it is also possible to download gene expression data sets in the same format. After loading these data sets in R, they can similarly be used within inSilicoMerging.

### Implementation of merging algorithms

A set of six batch effect removal methods for merging gene expression data is currently provided: BMC (Batch Mean-Centering [21]), COMBAT (Empirical Bayes [18]), DWD (Distance-Weighted Discrimination [17]), GENENORM (Z-score standardization), NONE and XPN (Cross-Platform Normalization [19]). For a detailed description of the major algorithms we refer to the original publications and in addition each of those six methods is also described in the accompanying package vignette. For more information about merging through batch effect removal in general please consult [22].

The implementation of COMBAT was based on the orginal R code made available by the authors on their website http://www.bu.edu/jlab/wp-assets/ComBat/Download∖_files/ComBat.R. The implementation of DWD uses the kDWD function from the corresponding package [23]. XPN has been implemented based on Matlab code provided by the authors https://genome.unc.edu/xpn/. The remaining merging algorithms were implemented using basic R functions. All implementations were tested by empirical validation and when possible, by comparing results with those from the original implementations.

Note that gene expression data sets from different platforms can be merged by keeping only the common features (genes or probe sets) in the merged data set. Some merging techniques are only reported and implemented to merge exactly two studies (e.g., XPN and DWD). To merge multiple studies, an additional step was added in which all studies were combined two-by-two in a recursive way.

### Implementation of validation algorithms

For the visual inspection of merging results, five qualitative validation methods are provided. In addition, six quantitative validation methods are provided as well. These quantitative indices provide a more accurate evaluation of the batch effect removal and they are very effective tools for comparing the results of different methods. Below a brief description of each validation method is provided, for an extensive survey we again invite the reader to consult [22].

#### Qualitative validation methods

Five qualitative validation methods are implemented:

##### plotMDS

creates a double-labeled Multidimensional Scaling (MDS) plot.

##### plotRLE

creates a relative log expression (RLE) plot, initially proposed to measure the overall quality of a data set [24] but also useful in this context.

##### plotGeneWiseBoxPlots

provides a local visualization by looking at the box plots of a specific gene across all samples.

##### plotDendrogram

creates a sample-wise hierarchical clustering plot.

##### plotGeneWiseDensity

is another local visualization tool which plots the estimated density of genes across different data sets.

If available we extended the relevant existing functions from R and Bioconductor.

#### Quantitative validation methods

Six quantitative validation indices were implemented, based on the description in [19]:

##### measureAsymmetry

This measure compares the sample asymmetry by calculating the skewness before and after batch effect removal. Good merging methods should not alter the samples’ skewness.

##### measureSamplesMeanCorrCoef

This measure compares the correlation between the same samples before and after batch effect removal. Good merging methods should maintain similarity and therefore correlation should be high.

##### measureGenesMeanCorrCoef

This measure compares the correlation between the same genes before and after batch effect removal. Good merging methods should maintain similarity and therefore correlation should be high.

##### measureSignificantGenesOverlap

This measure compares the number of known control genes found in the top ranked genes according to a particular method for differential expressed genes discovery in the adjusted study with those found in the original studies.

##### measureSamplesOverlap

This measure is an indication of how well the samples from each study are mixed after batch effect removal. For each array in each study the distance to the nearest array in all other studies is calculated. The average of these distances is used as a measure. The lower the average distance, the better the overlap, and the better the quality of the batch effect removal.

##### measureGenesOverlap

We also implemented a novel quantitative validation index which calculates the average difference in the distribution of all genes in the individual studies as follows:

(1) $$\mathrm{GO}V_{i}=\frac{\sum |P_{x_{i}}-P_{y_{i}}|}{2}$$

where $\left(P_{x_{i}}\right)$ and $\left(P_{y_{i}}\right)$ are the normalized probability density functions (such that $\left(\underset{i}{\sum }P_{x_{i}}=1\right)$ and $\left(\underset{i}{\sum }P_{y_{i}}=1\right)$) of gene *g*_*i*_ in the first respectively second data set and they are empirically estimated using the Parzen-Rosenblatt density estimation method [25]. Note that this index is bounded in [0 1], the minimum value being obtained when the two distributions are identical while the maximum value is reached when the two distributions are completely separated. The global cross-studies genes’ overlapping index is given by:

(2) $$\mathrm{GOV}=\frac{\overset{m}{\underset{i}{\sum }}\mathrm{GO}V_{i}}{m}$$

where *m* is the number of common genes between the two data sets. Note that *GOV * is still bounded in [0 1], providing a clear quantification of the batch effect or of the quality of the data integration process.

## Results and discussion

In the following sections we demonstrate the ease and power of the inSilicoMerging package based on an example analysis of two lung cancer data sets assayed on different platforms. We will discuss a complete workflow consisting of search and retrieval of data, followed by merging and corresponding validation.

### Accessing Data

We start our example analysis by using the inSilicoDb package to search for data sets containing lung cancer samples, assayed on two different platforms: Affymetrix Human Genome U133A Array (GPL96) and Affymetrix Human Genome U133 Plus 2.0 Array (GPL570). We restrict our search to data sets with the number of samples between 100 and 150 (the select function). All data sets retrieved from the InSilico DB database are consistently preprocessed using frozen RMA [26], as explained in [5].

> library("inSilicoDb");

> lung96 = getDatasetList("GPL96", "FRMA", query="lung cancer");

> lung570 = getDatasetList("GPL570", "FRMA", query="lung cancer");

> select = function(x, platform) {

+ n = nrow(pData(getAnnotations(x, platform)));

+ if(n > 100 & n < 150) { return(x); }

 + };

> lung96 = unlist(sapply(lung96, select, platform="GPL96"));

"GSE2603" "GSE10072" "GSE3218"

> lung570 = unlist(sapply(lung570, select, platform="GPL570"));

"GSE8332" "GSE19667" "GSE19804" "GSE19407" "GSE20257"

For both platforms we select one study from the above lists and retrieve the fully annotated and preprocessed data set:

> eset570 = getDataset("GSE19804", "GPL570", "FRMA", genes=TRUE);

> eset96 = getDataset("GSE10072", "GPL96", "FRMA", genes=TRUE);

> table(pData(eset570)[,"Disease"]);

control lung cancer

4958

> table(pData(eset96)[,"Disease"]);

control lung cancer

6060

Both studies contain normal and tumor samples with a common Disease annotation label and were selected for this example because they are almost equally divided in both disease subdivisions as can be seen in the previous code example.

### Batch Effect Removal and Merging

The different merging methods implemented in the inSilicoMerging package can now all be applied on the retrieved ExpressionSets by calling a single function merge. For example, simple combination without batch effect removal can be done as follows: > merge(esets, method="NONE");with esets a list of ExpressionSet objects. The method parameter can be one of the options described above: BMC, COMBAT, DWD, GENENORM, NONE or XPN.

Next to the merged set where no transformation (NONE) is applied and we also build a merged set using the COMBAT method, hence creating two different ExpressionSet objects containing merged data set:

> library(inSilicoMerging);

> esets = list(eset570, eset96);

> eset_NONE = merge(esets, method="NONE");

> eset_COMBAT = merge(esets, method="COMBAT");

> table(pData(eset_NONE)[,"Disease"])

control lung cancer

109118

### Validation

Five visual validation methods are provided by the package in order to immediately enable a first inspection of the merged data sets. For this example, a first global visual inspection of the two merging approaches applied is demonstrated via the plotMDS function.

**Figure 1:**
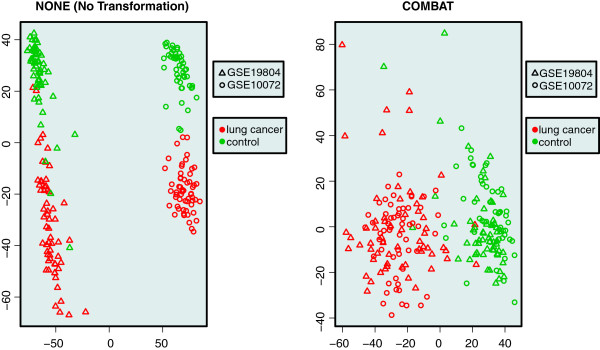
**MDS plots.** Visual inspection of two merged data sets using double-labeled Multi Dimensional Scaling (MDS) plots. In these MDS plots samples are labeled by color based on the target biological variable of interest and are labeled by symbol based on the study they originate from. On the left the two data sets are merged without any transformation and on the right the two data sets are merged by using the COMBAT method. It is intuitively clear from the MDS plots that samples cluster by study without any transformation and by disease after performing COMBAT.

> par(mfrow=c(1,2));

> plotMDS(eset_NONE,

+targetAnnot = "Disease",

+batchAnnot = "Study",

+main = "NONE (No Transformation)");

> plotMDS(eset_COMBAT,

+targetAnnot = "Disease",

+batchAnnot = "Study",

+main = "COMBAT");

In Figure 1 the two MDS plots are shown. Samples are labeled by color, based on the target biological variable of interest (normal versus lung cancer) and are labeled by symbol, based on the study they originate from. This information was retrieved from the curated phenotype information of the data sets using the targetAnnot and batchAnnot arguments, respectively. No extra manual step was required.

We can observe the effect of using COMBAT on the right part of Figure 1, where samples are more clustered together based on the target biological variable of interest (color), instead of the study they originate from (symbol). On the left part, if no merging technique is applied, a huge inter-study bias can be observed, hindering any further analysis of this combined data set.

Similarly, on a local scale we demonstrate the use of the plotGeneWiseDensity function for a specific gene.

**Figure 2:**
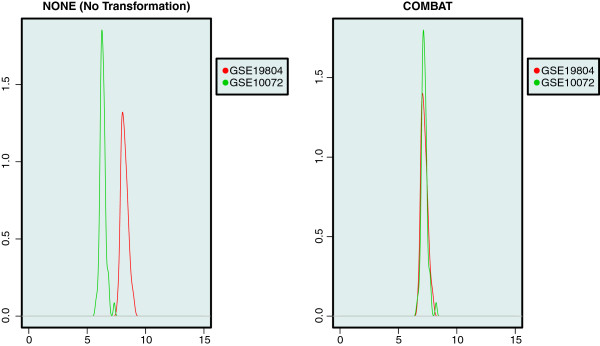
**Genewise density plots.** Visual inspection of two merged data sets using gene-wise density plots. For the randomly selected MYL4 gene, density plots in each study are shown, colored by study. On the left the two data sets are merged without any transformation and on the right the two data sets are merged by using the COMBAT method. The genewise density plots show that after transformation the distribution is much more similar.

> par(mfrow=c(1,2));

> plotGeneWiseDensity(eset_NONE,

+batchAnnot = "Study",

+gene = "MYL4",

+main = "NONE (No Transformation)");

> plotGeneWiseDensity(eset_COMBAT,

+batchAnnot = "Study",

+gene = "MYL4",

+main = "COMBAT");

Gene MYL4 was arbitrary selected and its estimated density across both studies is shown in Figure 2. We can observe that the COMBAT method makes the distributions of this specific gene across the two studies more similar to each other. Note that the magnitude of the batch effect at the gene level is very dependent on the specific gene selected.

**Figure 3:**
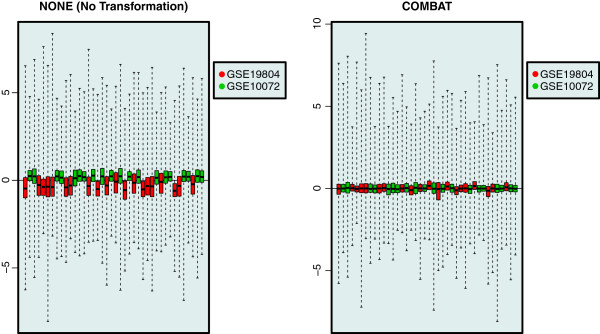
**RLE plots.** Visual inspection of two merged data sets using relative log expression plots. In these relative log expression plots samples are colored by study. For clarity purposes only 40 randomly selected samples are shown. On the left the two data sets are merged without any transformation and on the right the two data sets are merged by using the COMBAT method. After applying COMBAT the mean of the RLE is approximately 0 for all genes which indicates a good batch effect removal.

**Figure 4:**
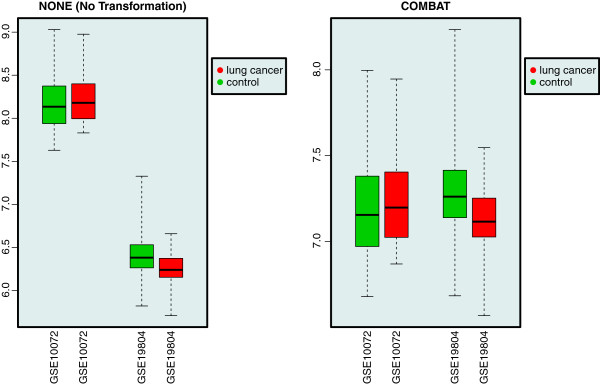
**Genewise box plots.** Visual inspection of two merged data sets using a gene-wise box plots. Boxplots of the randomly selected MYL4 gene are grouped by study and colored by the target biological variable of interest. On the left the two data sets are merged without any transformation and on the right the two data sets are merged by using the COMBAT method. After batch effect removal the distribution of the gene is much more similar between studies than without.

**Figure 5:**
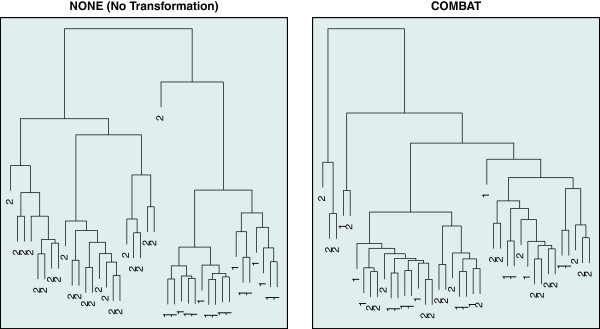
**Dendrogram plots.** Visual inspection of two merged data sets using dendrograms plots. In these dendrogram plots samples are labeled by a number corresponding to the study they originate from. For clarity purposes only 40 randomly selected samples are used to perform the hierarchical clustering. On the left the two data sets are merged without any transformation and on the right the two data sets are merged by using the COMBAT method. In the right plot it can be seen that samples originating from different studies are mixed, while on the left they are grouped per study.

R code illustrating the five visual inspection methods is provided in the Additional file 1. The results for the three remaining methods can be found in Figures 3, 4, 5.

### Comparison with existing tools

We compare inSilicoMerging, together with inSilicoDb, on three axes: data access, data merging and validation algorithms.

#### Data access

GEOQuery [27] is an existing package to retrieve gene expression data sets in R/Bioconductor. However, the information about the samples is in a raw form requiring a manual curation step in transit between a data repository (e.g., GEO) and a data analysis platform (e.g., R/Bioconductor). A lack of standardized phenotypic meta-data and inconsistent preprocessing can hinder the integration and combination of different raw form data sets.

#### Data merging

Several of the batch effect removal techniques included in the inSilicoMerging package have been implemented prior to this package in different programming and/or script languages (e.g., R, Matlab, Java). A major advantage of inSilicoMerging is the consistent interface for all merging methods and its integration in the R/Bioconductor framework. We will briefly discuss the added value of our package compared to those already available in R.

The authors of the COMBAT method provide an implementation on their website which allows the input of known covariates (besides batch id). They work with several matrices containing the numerical data, and relevant annotations and covariates. Within our package, all this information is bundled and encoded in the ExpressionSet structure.

The authors of the DWD method recently have released the DWD R/Bioconductor package [23]. This package is intended as a general use of the DWD technique and is not specific for the goal of batch effect removal. It is therefore not straightforward to use as a merging technique for gene expression data sets and once again the relevant data is dispersed over several objects. In addition, the necessary transformation of the gene expression values was added to the inSilicoMerging package in order to obtain a merged data set as result.

The CONOR package which is available through the CRAN repository [28], most closely resembles our software as it includes multiple batch effect removal methods and several methods based on discretizing the gene expression data. However, it lacks the user friendliness provided by our package and the direct integration with the Bioconductor framework.

#### Validation

Evaluating and validating the results of batch effect removal methods is perhaps as important and difficult as the batch effect removal process itself. Without good and reliable evaluation tools, these methods could result in an increased distortion of the data, introducing serious errors in the results of any downstream analysis performed.

To the best of our knowledge no explicit software has been developed for validating the quality of the batch effect removal process of microarray gene expression data sets. The collection of both qualitative and quantitative validation methods is therefore a unique property of the inSilicoMerging package.

## Conclusions

The inSilicoMerging R/Bioconductor package enables the integration of batch adjustment in any workflow dealing with large-scale analysis of gene expression data, paving the road towards unlocking the potential of the massive amount of publicly available microarray data. Moreover, its seamless integration with the R/Bioconductor framework for further analysis and the inSillicoDb package for retrieval of consistent data, minimizes the need for manual intervention. As a result transparency, trackability and reproducibility of large-scale gene expression analysis is significantly increased.

## Availability and requirements

**Project name:**inSilicoMerging**Project home page:**http://www.bioconductor.org/packages/release/bioc/html/inSilicoMerging.html**Operating system(s):** Platform independent**Programming language:** R**Other requirements:** Bioconductor**License:** GPL-2**Any restrictions to use by non-academics:** none

## Endnote

^1^http://www.insilico.ulb.ac.be

## Competing interests

The authors declare that they have no competing interests.

## Author’s contributions

JT, SM and CL designed and implemented the inSilicoMerging package and drafted the manuscript. JT, DS and RD designed and implemented the inSilicoDb package. AC, CM and DWS were involved in the development of the back-end software. VdS provided the expert-curated annotation data of the underlying database tool. HB and AN conceived the study and participated in its design and coordination. All authors read and approved the final manuscript.

## Supplementary Material

Additional file 1 R script. code_example.R R script using both inSilicoDb and inSilicoMerging packages generating Figure 1-5. Click here for file
